# Cannabidiol (CBD): a killer for inflammatory rheumatoid arthritis synovial fibroblasts

**DOI:** 10.1038/s41419-020-02892-1

**Published:** 2020-09-01

**Authors:** Torsten Lowin, Ren Tingting, Julia Zurmahr, Tim Classen, Matthias Schneider, Georg Pongratz

**Affiliations:** 1 Poliklinik, Funktionsbereich & Hiller Forschungszentrum für Rheumatologie, University Hospital Duesseldorf, D-40225 Duesseldorf, Germany; 2 Klinik für Orthopädie/Orthopädische Rheumatologie, St. Elisabeth-Hospital Meerbusch-Lank, D-40668 Meerbusch, Germany

**Keywords:** Immune cell death, Rheumatoid arthritis

## Abstract

Cannabidiol (CBD) is a non-intoxicating phytocannabinoid from *cannabis sativa* that has demonstrated anti-inflammatory effects in several inflammatory conditions including arthritis. However, CBD binds to several receptors and enzymes and, therefore, its mode of action remains elusive. In this study, we show that CBD increases intracellular calcium levels, reduces cell viability and IL-6/IL-8/MMP-3 production of rheumatoid arthritis synovial fibroblasts (RASF). These effects were pronounced under inflammatory conditions by activating transient receptor potential ankyrin (TRPA1), and by opening of the mitochondrial permeability transition pore. Changes in intracellular calcium and cell viability were determined by using the fluorescent dyes Cal-520/PoPo3 together with cell titer blue and the luminescent dye RealTime-glo. Cell-based impedance measurements were conducted with the XCELLigence system and TRPA1 protein was detected by flow cytometry. Cytokine production was evaluated by ELISA. CBD reduced cell viability, proliferation, and IL-6/IL-8 production of RASF. Moreover, CBD increased intracellular calcium and uptake of the cationic viability dye PoPo3 in RASF, which was enhanced by pre-treatment with TNF. Concomitant incubation of CBD with the TRPA1 antagonist A967079 but not the TRPV1 antagonist capsazepine reduced the effects of CBD on calcium and PoPo3 uptake. In addition, an inhibitor of the mitochondrial permeability transition pore, cyclosporin A, also blocked the effects of CBD on cell viability and IL-8 production. PoPo3 uptake was inhibited by the voltage-dependent anion-selective channel inhibitor DIDS and Decynium-22, an inhibitor for all organic cation transporter isoforms. CBD increases intracellular calcium levels, reduces cell viability, and IL-6/IL-8/MMP-3 production of RASF by activating TRPA1 and mitochondrial targets. This effect was enhanced by pre-treatment with TNF suggesting that CBD preferentially targets activated, pro-inflammatory RASF. Thus, CBD possesses anti-arthritic activity and might ameliorate arthritis via targeting synovial fibroblasts under inflammatory conditions.

## Introduction

Cannabidiol (CBD) is a non-intoxicating cannabinoid found in *cannabis sativa*^1^. In contrast to the psychoactive constituent tetrahydrocannabinol (THC), CBD demonstrates no direct effect at cannabinoid receptors 1 and 2 (CB_1_ and CB_2_) but modulates the effect of agonists suggesting an allosteric function^2^. In addition, CBD binds to PPARγ, GPR3/6/12/18/55, TRPV1/2, TRPA1, 5-hydroxytryptamine receptor, and mitochondrial proteins^3–11^. Despite its promiscuous pharmacology, CBD is well tolerated even when given in high concentrations^12,13^. Side effects of CBD in humans include diarrhea and fatigue and, more importantly, CBD interacts with other drugs since it is metabolized by CYP enzymes in the liver thereby inhibiting the degradation of other therapeutic compounds^14,15^. While the therapeutic benefits of CBD in childhood epilepsy are well documented, its effects on inflammation have only been investigated in animal models^13,16^. Studies in rodents with osteoarthritis or collagen-induced arthritis demonstrated anti-inflammatory and analgesic effects of CBD, but these studies did not identify the mechanism of action^17–19^. Here, we investigate the effect of CBD on intracellular calcium, cell viability, and cytokine production in rheumatoid arthritis synovial fibroblasts (RASF). RASF are one major contributor of joint destruction in RA as they secrete pro-inflammatory cytokines and matrix degrading enzymes^20^. In fact, subsets of RASF selectively mediate joint destruction or the inflammatory response, emphasizing their important role in the pathogenesis of RA^21^. In previous studies, we already identified TRPA1 as a therapeutic target since the TRPA1 agonist Polygodial selectively deleted TNF-activated RASF^22^. CBD also binds TRPA1^7,23^, and therefore we hypothesized that CBD has detrimental effects on cell viability, which might explain in part its mechanism of action at sites of inflammation.

## Results

### CBD reduces cell viability and proliferation of RASF

**Fig. 1:**
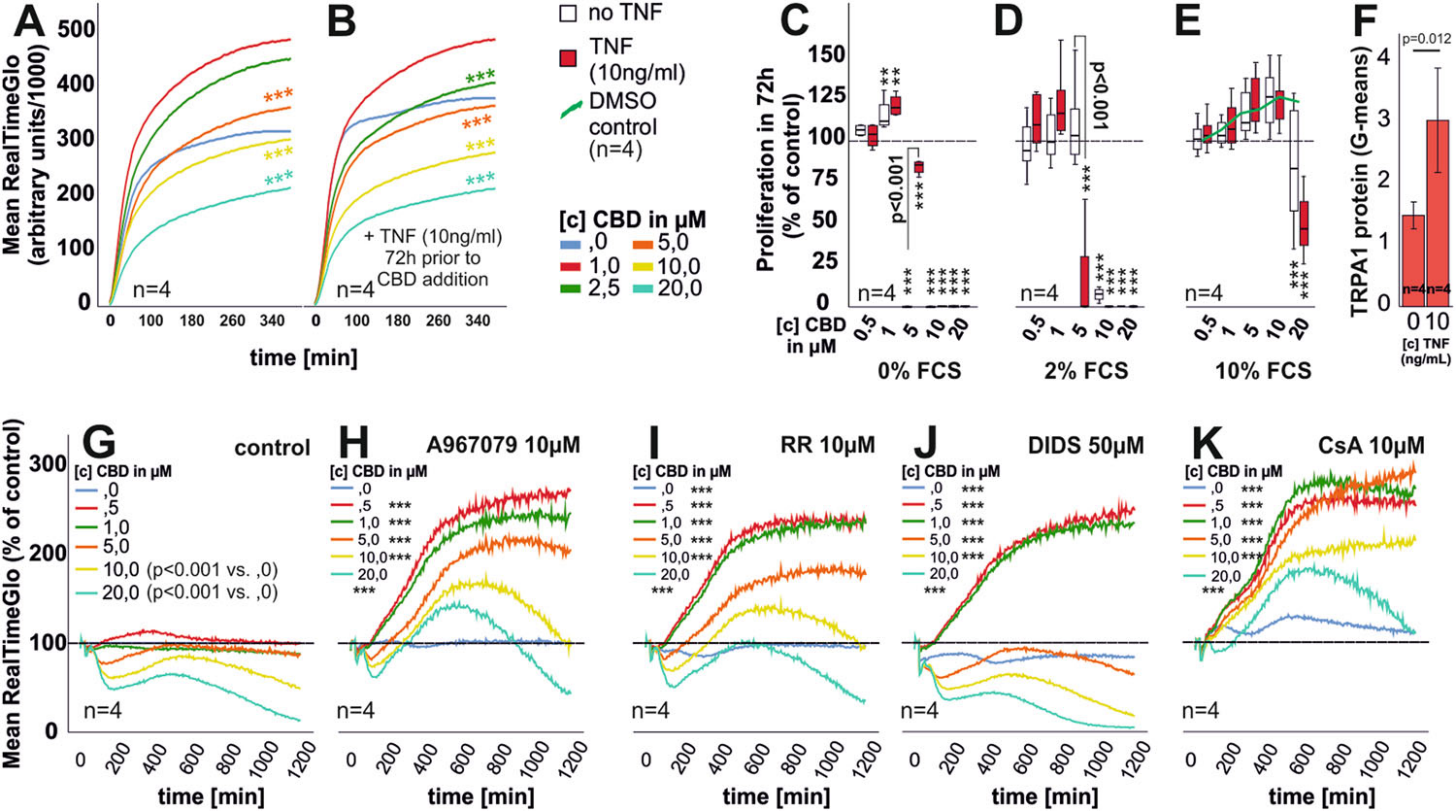
Assessment of cell viability, TRPA1 expression, and RASF proliferation. **a**, **b** Mean cell viability of unstimulated (**a**) or TNF pre-stimulated (**b**) RASF after CBD challenge monitored in real-time over the course of 375 min. **c**–**e** Mean proliferation of RASF with (red bars) and without (white bars) TNF pre-stimulation in response to CBD in medium containing 0% FCS (**c**), 2% FCS (**d**), and 10% FCS. The dotted line represents the unstimulated control, which was set to 100%. **f** Flow cytometric detection of TRPA1 protein in RASF with or without TNF stimulation for 72 h. **g**–**k** Mean cell viability of TNF pre-stimulated RASF after CBD challenge and concomitant addition of inhibitors over the course of 20 h. *n* is the number of replicates and patient samples investigated. ANOVA with Dunnett’s T3 post-hoc test was used for comparisons in **a**, **b**, **g**–**k**. ANOVA with Bonferroni post-hoc test was used for comparisons in **c**–**e**. Two-tailed *t*-test was used for comparisons in **f**. *p < 0.05; **p < 0.01, ***p < 0.001 vs control. The error bars in **c**–**f** represent the standard error of mean (sem).

Over the course of 6 h we found that CBD (≥5 µM) decreases cell viability (Fig. 1a, b), but a stimulatory effect was detected for 1 µM CBD in TNF pre-incubated RASF (Fig. 1b). CBD combined with the TRPA1 antagonist, A967079, recovered cell viability (Fig. 1h). TRPA1 is upregulated by TNF (Fig. 1f) and we also detected an increase of TRPA1 mRNA by real-time PCR after 24 h under the influence of TNF (data not shown). Ruthenium Red (RR) also reduced the detrimental effects of CBD (Fig. 1i). Surprisingly, 4,4′-Diisothiocyanatostilbene-2,2′-disulfonate (DIDS), supported cell viability at low CBD concentrations but enhanced its cytotoxic effects at concentrations ≥1 µM (Fig. 1j), while Cyclosporin A (CsA), blocked the effects of CBD (Fig. 1k). Since RealTime-Glo assays were conducted at 37 °C in serum-free medium but without CO_2_ and humidity control, we also confirmed these results in cell titer blue endpoint assays (Fig. 5d). In vivo, CBD is bound to lipoproteins/albumin lowering the available concentration of free CBD^24^. Therefore, we investigated the effect of fetal calf serum content with CBD on proliferation. CBD in concentrations ≥5 µM reduced proliferation of RASF in medium without or 2% FCS (Fig. 1c, d). TNF pre-stimulation enhanced proliferation at 5 µM CBD in 0% FCS (Fig. 1c) while the opposite was true using 2% FCS (Fig. 1d). With 10% FCS, CBD inhibited proliferation of RASF only at 20 µM (Fig. 1e). DMSO (vehicle control) alone had a stimulatory effect on proliferation (Fig. 1e, green line). These findings underline the need for relatively high concentrations of CBD when used in in vivo settings^13^.

### CBD increases intracellular calcium and its effects are enhanced by TNF

**Fig. 2:**
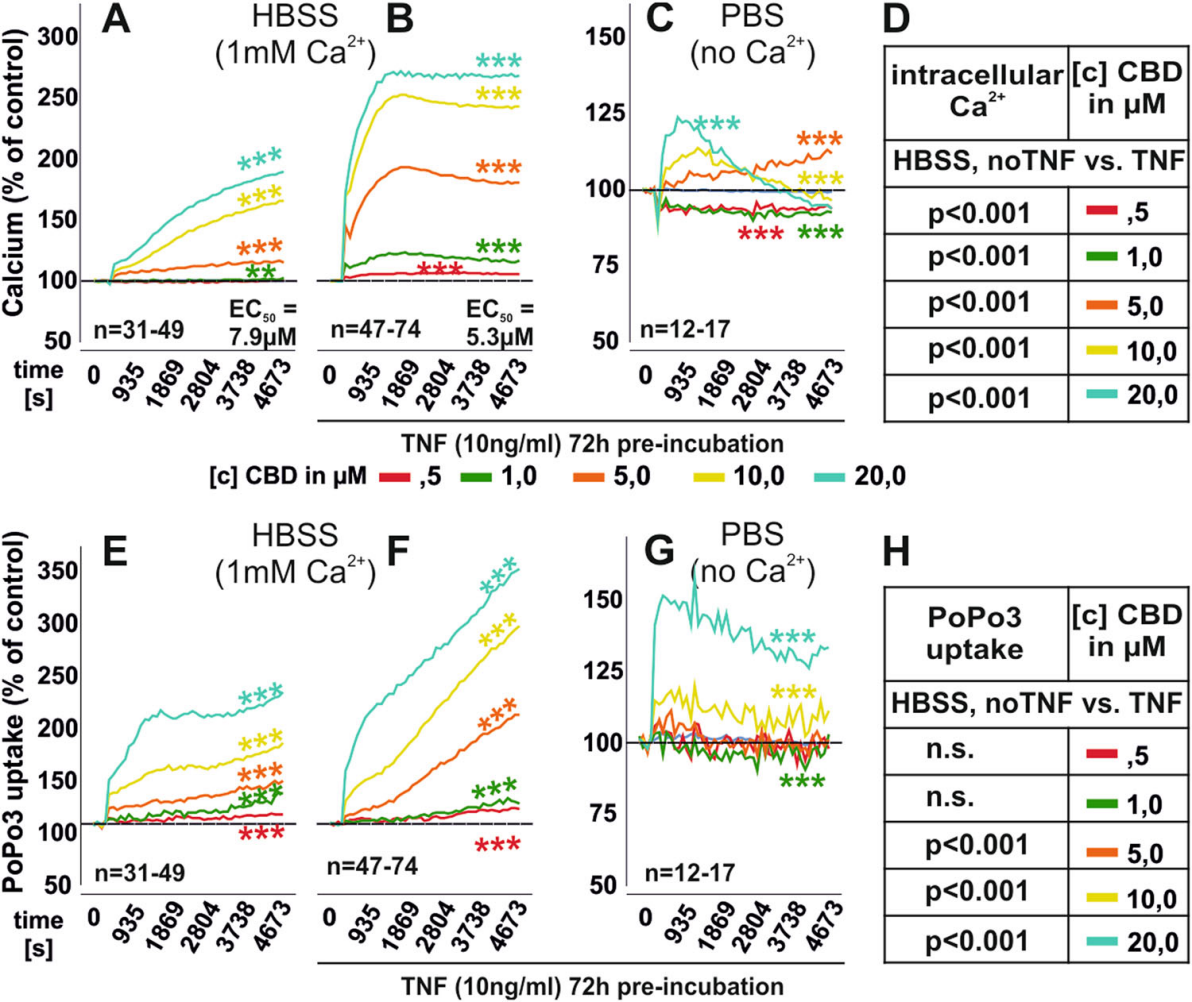
Mean intracellular calcium levels and PoP3 uptake of RASF in response to CBD. **a**–**c** Intracellular calcium mobilization of RASF after CBD challenge in HBSS (**a**, **b**) or without extracellular Ca^2+^ (PBS; **c**). The EC_50_ values obtained for the increase of intracellular calcium were significantly different (*p* < 0.001) between unstimulated and TNF-pre-stimulated RASF. **e**–**g** PoPo3 uptake by RASF after CBD challenge in HBSS (**e**, **f**) or without extracellular Ca^2+^ (PBS; **f**). RASF were pre-stimulated with TNF (**b**, **c**, **f**, **g**) or untreated (**a**, **e**). **d**, **h** Comparison between unstimulated and TNF pre-treated RASF regarding intracellular calcium levels (**d**) and PoPo3 uptake (**h**). *n* is the number of experiment replicates from 29 different patient samples (**a**, **e**), 38 patient samples (**b**, **f**), and 13 patient samples (**c**, **g**). ***p < 0.001, *p < 0.05 for differences between [c] of CBD. ANOVA with Dunnett’s T3 post-hoc test was used for all comparisons.

CBD influences calcium mobilization^9,23,25^ and we found that concentrations ≥5 µM increased intracellular calcium (Fig. 2a). The potency of CBD was enhanced by pre-incubation with TNF for 72 h (Fig. 2b). Under these conditions, intracellular calcium levels were significantly increased compared to untreated RASF (Fig. 2d). When extracellular calcium was omitted by using PBS, CBD still increased intracellular calcium although to a smaller extent (Fig. 2c) Besides calcium, we also analyzed the uptake of the cell viability dye PoPo3 iodide under CBD stimulation. PoPo3 uptake increases when membrane integrity is compromised during apoptosis or necrosis. CBD dose-dependently increased the uptake of PoPo3 which was enhanced by extracellular calcium and TNF pre-stimulation (Fig. 2f, g). Basal uptake of PoPo3 and intracellular calcium levels were increased by TNF pre-treatment (Supplementary Fig. 3A, B). The detrimental effect of CBD on cell viability was also confirmed in the XCELLigence system using untreated RASF (Supplementary Fig. 1).

### Calcium mobilization and PoPo3 uptake partly depend on TRPA1 activation

**Fig. 3:**
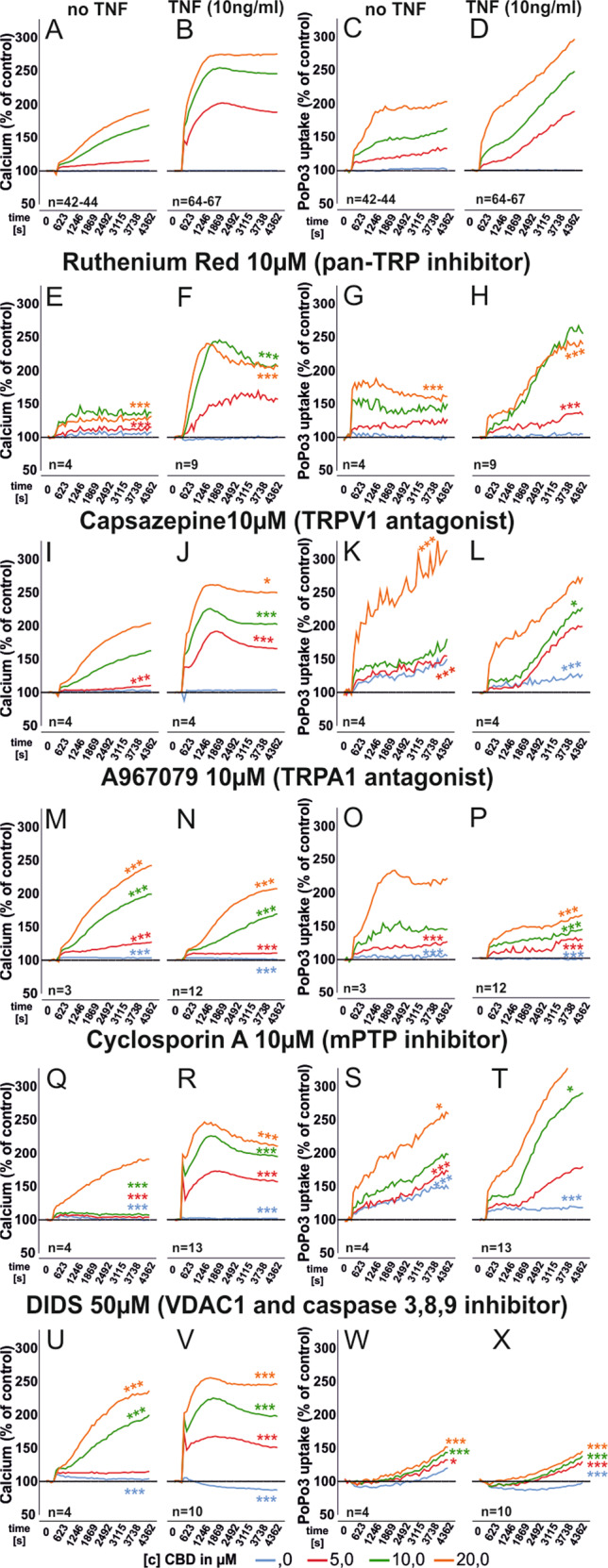
Modulation of intracellular calcium levels and PoP3 uptake of RASF after CBD challenge with concomitant inhibition of membrane TRP channels and mitochondrial targets. Mean intracellular calcium mobilization (**a**, **b**, **e**, **f**, **i**, **j**, **m**, **n**, **q**, **r**, **u**, **v**) and mean PoPo3 uptake (**c**, **d**, **g**, **h**, **k**, **l**, **o**, **p**, **s**, **t**, **w**, **x**) of RASF after CBD challenge and concomitant inhibition with the antagonists given in the figure. *n* is the number of experiment replicates from 24 patient samples (**a**, **c**), 33 patient samples (**b**, **d**), and 8 patient samples (**n**, **p**). **e**–**x** (except **n**, **p**) *n* number equals number of replicates and different patient samples. ANOVA with Dunnett’s T3 post-hoc test was used for all comparisons versus (**a**–**d**). *p < 0.05, ***p < 0.001.

Since CBD binds TRPV1, TRPV2, and TRPA1^7^ we investigated the involvement of these ion channels. RR, a general inhibitor for several TRP channels^26–28^, reduced intracellular calcium levels (Fig. 3e, f) and PoPo3 uptake (Fig. 3g, h) but the magnitude of inhibiton was small. RR did not change basal calcium levels or PoPo3 uptake in unstimulated but did so in TNF pre-stimulated RASF (Supplementary Fig. 3). Next, we combined CBD with the TRPV1 antagonist Capsazepine (CPZ)^29^. CPZ had only a minor influence on CBD-induced calcium levels and PoPo3 uptake (Fig. 3i–l). Of note, CPZ also modulated basal calcium and PoPo3 levels (Supplementary Fig. 3) With TRPA1 inhibition we found that without TNF pre-stimulation, the antagonist A967079 (10 µM) increased intracellular calcium (Fig. 3m) but decreased it when RASF were pre-incubated with TNF (Fig. 3n). A967079 increased PoPo3 uptake under basal conditions (Fig. 3o) but decreased it after TNF pre-incubation (Fig. 3p). Furthermore, A967079 enhanced basal calcium levels and PoPo3 uptake (Supplementary Fig. 3A–D).

### Mitochondrial targets mediate the effects of CBD

**Fig. 4:**
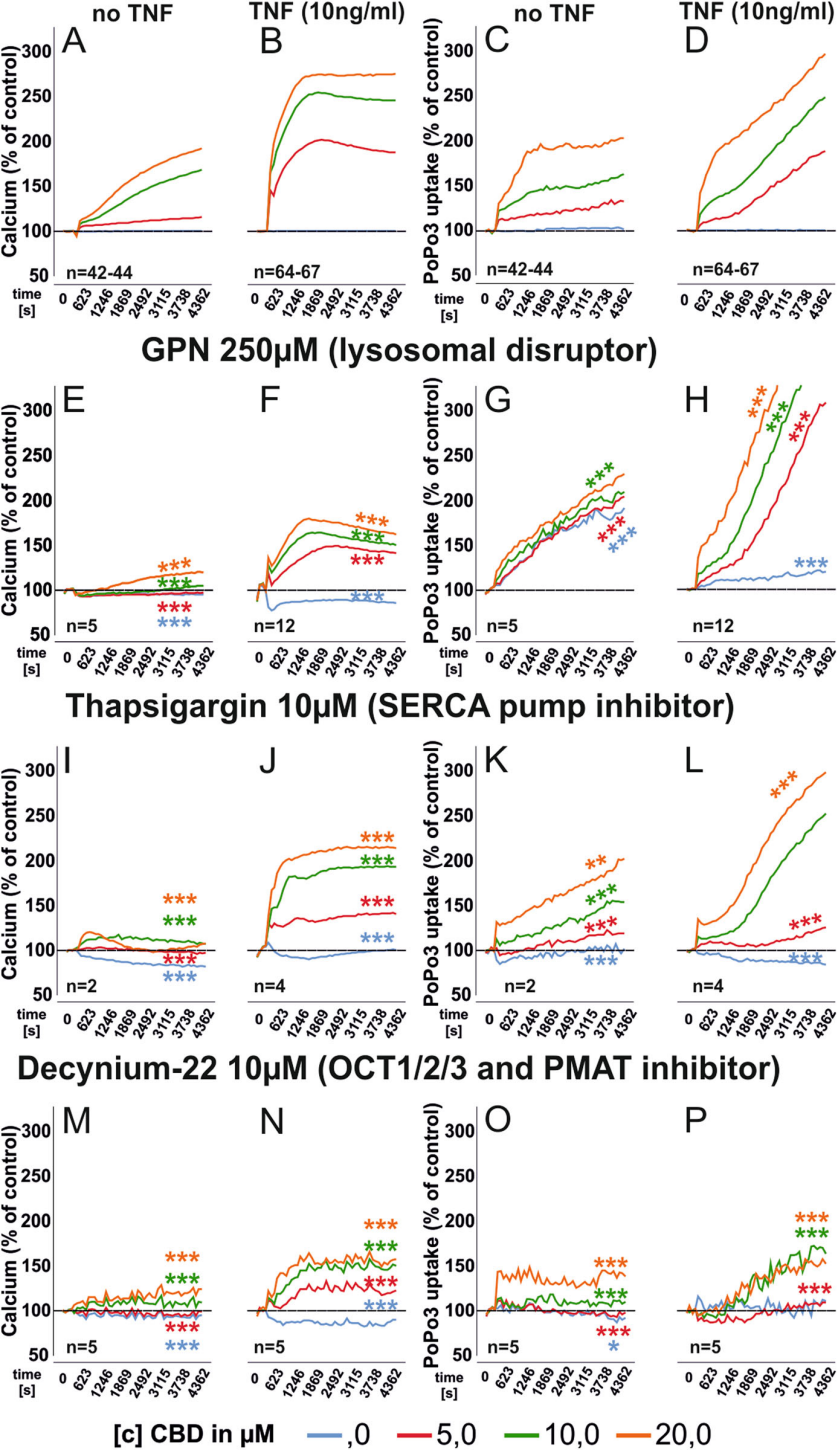
Modulation of intracellular calcium levels and PoP3 uptake of RASF by CBD with concomitant manipulation of lysosomal and endoplasmatic reticulum calcium stores or inhibition of organic cation transport. Mean intracellular calcium mobilization (**a**, **b**, **e**, **f**, **i**, **j**, **m**, **n**) and mean PoPo3 uptake (**c**, **d**, **g**, **h**, **k**, **l**, **o**, **p**) of RASF after CBD challenge and concomitant inhibition with the antagonists given in the figure. ANOVA with Dunnett’s T3 post-hoc test was used for all comparisons. ***p < 0.001.

**Fig. 5:**
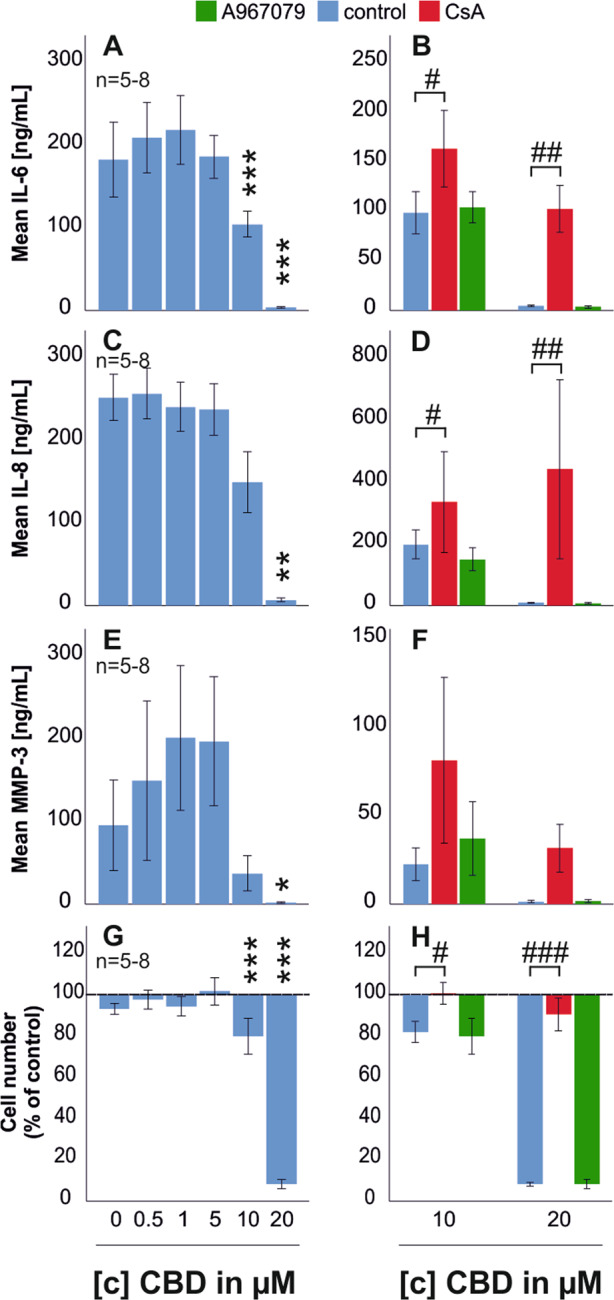
IL-6, IL-8, MMP-3 production, and cell number after 72 h incubation with CBD. RASF were incubated for 72 h with TNF. After wash-off, RASF were challenged with antagonists for 30 min followed by CBD addition for 72 h. ANOVA was used for all comparisons vs control w/o CBD. **a**, **c**, **e**, **g** IL-6, IL-8, MMP-3 production, and cell number after 72 h challenge with CBD. **b**, **d**, **f**, **h** IL-6, IL-8, MMP-3 production, and cell number after 72 h challenge with CBD (10 µM and 20 µM) with concomitant addition of the TRPA1 antagonist A967079 and the mPTP inhibitor CsA. Significant differences between CBD in different concentrations are depicted as *p < 0.05, **p < 0.01, and ***p < 0.001, and CBD versus CBD/antagonist treatment are depicted as #*p* < 0.05, ##*p* < 0.01, and ###*p* < 0.001. ANOVA with Bonferroni post-hoc test was used for all comparisons. The dotted line in **g**, **h** represents the control value which was set to 100%. *n* is the number of replicates out of four different patient samples. The error bars represent the standard error of mean.

We investigated four proposed mitochondrial targets for CBD: the mitochondrial calcium uniporter (MCU), the sodium/calcium exchanger (NCLX)^30^, the voltage-gated anion channel (VDAC1)^8^ and the mitochondrial membrane permeability transition pore (mPTP) which initiates apoptotic and necrotic events^31^.While we detected a minor influence of NCLX and MCU (Supplementary results and Supplementary Fig. 2) on intracellular calcium levels and PoPo3 uptake, inhibition of mPTP exerted the strongest influence (Fig. 3q–t). CBD-induced changes in intracellular calcium were reduced by the mPTP inhibitor CsA (Fig. 3q). In TNF pre-stimulated RASF, CsA reduced calcium levels over all CBD concentrations (Fig. 3r). In addition, CsA accelerated PoPo3 uptake (Fig. 3s, t) but did not alter basal calcium or PoPo3 levels (Supplementary Fig. 3A–D). Next, we combined CBD with DIDS, which is an inhibitor of VDAC in the outer mitochondrial and the plasma membrane^32,33^. CBD stabilizes a closed conformation of VDAC, which excludes the exchange of metabolites from the cytosol into mitochondria, but enhances its calcium transport function^8,34^. DIDS increased CBD-induced calcium mobilization regardless of TNF pre-stimulation (Fig. 3u, v) and it completely abolished PoPo3 uptake under all conditions (Fig. 3w, x). DIDS also increased basal calcium levels in TNF pre-stimulated RASF (Supplementary Fig. 3B) and decreased PoPo3 levels (Supplementary Fig. 3C, D). Since we found TRPA1 to be involved in the effects of CBD (Figs. 1h and 3m–p), we were interested in the cellular localization of this receptor. Since we assumed this to be an intracellular site, we used Thapsigargin to deplete calcium stores in the endoplasmatic reticulum (ER)^35,36^ and Gly-Phe-β-naphthylamide (GPN), which disrupts lysosomes and releases calcium stored in this organelle^37,38^. We found that GPN reduced the elevation of intracellular calcium by CBD (*p* < 0.001, Fig. 4e, f), while PoPo3 uptake was significantly enhanced (Fig. 4g, h). Of note, GPN per se induced an increase of intracellular calcium and PoPo3 uptake (Supplementary Fig. 3A–D). Similarly, the inhibitor of the endoplasmic reticulum Ca^2+^-ATPase, Thapsigargin, reduced the CBD-induced increase in intracellular calcium levels (Fig. 4i, j) and slightly attenuated PoPo3 uptake (Fig. 4k, l) but increased basal calcium and PoPo3 levels (Supplementary Fig. 3A–D). Cationic and uncharged compounds are taken up by organic cation transporters (OCT)^39,40^ and therefore we assessed the effects of Decynium-22 (D22), an inhibitor of all OCT isoforms on intracellular calcium and the uptake of PoPo3. Under all conditions, we found that D22 prevented the increase of intracellular calcium/PoPo3 uptake induced by CBD (Fig. 4m–p) and it reduced basal intracellular calcium (Supplementary Fig. 3A, B) but slightly elevated PoPo3 levels (Fig. 3c, d). Lastly, we determined the influence of CBD on the production of IL-6, IL-8, and MMP-3 by RASF. CBD (10 µM and 20 µM) significantly decreased the production of IL-6 (Fig. 5a), which was inhibited by the addition of CsA (Fig. 5b). IL-8 production was modified by CBD (20 µM) alone (Fig. 5c) and the addition of CsA increased IL-8 levels significantly (Fig. 5d). MMP-3 levels were reduced by 20 µM CBD (Fig. 5e). The cytokine-reducing effects of CBD might be related to the reduction in viable cells, since we detected a reduction in cell number at 10 µM and 20 µM CBD which was inhibited by the addition of CsA (Fig. 5g, h).

## Discussion

In this study, we demonstrated that CBD decreases cell viability, proliferation, and cytokine production but increases intracellular calcium and PoPo3 levels of RASF and all effects were enhanced by TNF pre-stimulation. These effects were mediated by TRPA1 and by the assembly of the mPTP under pro-inflammatory conditions, whereas under unstimulated conditions, TRPA1 was not involved.

We demonstrated that CBD reduces cell viability, but RealTime-Glo assays were conducted in serum-free medium without carrier protein. Therefore, we assessed whether CBD influences RASF proliferation in medium containing FCS, since in vivo, CBD is bound to serum albumin, which lowers its free concentration available for receptor binding^24,41^. We confirmed that the anti-proliferative effect of CBD is dependent on FCS content. Consequently, for in vivo applications, CBD needs to be administered in high concentrations to elicit beneficial effects as shown in the treatment of Dravet syndrome^13^. In order to identify the cellular targets for CBD, we used the TRPA1 antagonist A967079 and the pan-TRP antagonist RR to inhibit the effects of CBD on cell viability as CBD has already been identified as ligand for TRPA1^23^. Moreover, we found that CsA reversed the detrimental effects of CBD on cell viability, which confirms results from Olivas-Aguirre et al.^25^ that showed mitochondrial calcium overload correlates with assembly of the mPTP by CBD. It has been demonstrated that CBD influences calcium homeostasis^25^ and we also found CBD to elevate intracellular calcium in RASF. This confirms own previous results demonstrating calcium mobilization in response to TRPA1 ligation^22^. Moreover, we showed that TNF up-regulates TRPA1 protein in RASF, which translates into increased sensitivity to TRPA1 ligands^22^. CBD also increased calcium levels without extracellular calcium by using PBS instead of HBSS, suggesting mobilization form intracellular stores. In fact, in dorsal root ganglia neurons it has been shown that TRPA1 is located in lysosomes, where its activation fosters neurotransmitter release^42^. Although we do not provide direct evidence regarding the localization of TRPA1, the use of the cell-impermeable pan-TRP inhibitor RR^43^, which was only able to slightly attenuate the effects of CBD suggests an intracellular target protein. In line with this, the lipophilic antagonist A967079 decreased calcium mobilization and PoPo3 uptake after CBD challenge. Moreover, we used Thapsigargin to deplete ER calcium stores and GPN to disrupt lysosomes and both compounds reduced the elevation of intracellular calcium after CBD exposure. This shows that ER calcium stores are involved and it has been shown that even if calcium originates from lysosomes, the signal is amplified by depletion of ER stores^44^. This is important, because although GPN has been reported to mobilize lysosomal calcium, a recent study claimed that GPN increases calcium through an ER-dependent mechanism^45^. CBD is also an agonist at TRPV1/2 ion channels^7,23,46,47^, but neither CPZ nor RR inhibited the effects of CBD, ruling out these receptors as target molecules. CPZ is also an agonist at TRPA1^48^, and we did detect a small increase in basal intracellular calcium in response to this ligand alone. Accordingly, CPZ slightly elevated the calcium response of RASF to CBD suggesting a sensitizing effect on TRPA1. TRPA1 inhibition with A967079 reduced calcium mobilization and PoPo3 uptake in TNF pre-stimulated but not naïve RASF, where we found the opposite, suggesting that CBD exerts additional effects via different cellular targets besides TRPA1. This demonstrates that TRPA1 contributes to the rise in intracellular calcium only in TNF pre-stimulated RASF, in which TRPA1 is upregulated. Besides binding to TRPs, it has been demonstrated that CBD ligates several proteins^11,49^ with mitochondrial targets being the most prominent^8–10,25,30^. In mitochondria, CBD targets VDAC1, NCLX, MCU, and controls assembly of the mPTP^8–10,25^. Although protective effects of CBD against mitochondrial toxins have been shown^10,50^, the majority of studies demonstrated that CBD induces cell death by disturbing calcium homeostasis^8,9,25^. This confirms our results, since CBD augmented calcium levels with a concomitant increase in cell death. Excess cytosolic calcium is taken up by mitochondria which are depolarized in this process^51^. If mitochondrial calcium levels exceed a certain threshold, mPTP is assembled leading to cell death^52^. In fact, we demonstrated that only CsA prevented cell death, suggesting that mitochondrial calcium overload occurs in CBD-stimulated RASF. In line with this, CsA reduced intracellular calcium, which is another indicator that mitochondria provide a significant contribution to the increase in calcium by CBD. Calcium is increased by mPTP formation as mitochondria are permeabilized releasing stored calcium into the cytosol^53^. Thus, inhibiting mPTP formation by CsA decreases calcium leakage from mitochondria and subsequent cell death. In addition, we found that the NCLX inhibitor CGP reduced cytosolic calcium levels. CGP blocks calcium transport from the mitochondrial matrix into the intermembrane space, thus increasing mitochondrial and reducing cytosolic calcium levels^54^. We also used the reverse mode inhibitor of the NCLX, KB-R7943, but results with this inhibitor are difficult to interpret due to its interaction with the MCU and NCLX. In the latter it can act as forward or reverse mode antagonist dependent on cell type, NCLX isoform, and concentration^55,56^. Using the specific MCU inhibitor DS16570511 we found increased cytosolic calcium levels in TNF pre-stimulated but not in unstimulated RASF. This might be explained by TRPA1, which only contributed to calcium level alterations in TNF pre-treated RASF. For the inhibition of VDAC1 we used DIDS which increased intracellular calcium in unstimulated, but decreased calcium in TNF pre-treated RASF. This might depend on the initial calcium signal generated by CBD, because DIDS can permeabilize the inner mitochondrial membrane depending on calcium concentration leading to formation of mPTP^57^. Besides calcium, PoPo3 uptake served as readout for changes in cell viability as it is supposed to enter cells with a compromised plasma membrane only. However, several studies showed that the uptake of PoPo3 related compounds also occurs via specific receptors/ion channels^58,59^. In addition, it has been demonstrated that the family of organic cation transporters (OCT) mediates the uptake of many charged but also electroneutral compounds into the cell^39^. Therefore, it is quite possible that PoPo3 is also taken up by OCT and indeed we show that decynium-22, which inhibits all OCT isoforms^39^ strongly reduced PoPo3 uptake and it also blunted the increase of intracellular calcium, which might be due to the electrogenic properties of D22^40^. Another possibility is that OCT mediates the uptake of CBD and D22 would limit the access of CBD to intracellular compartments. D22 did not influence basal uptake of PoPo3, but reduced the CBD-induced uptake and this might be related to changes in intracellular calcium since the activity of OCT is regulated by calcium-dependent proteins^39^. DIDS completely blocked PoPo3 uptake but these results are difficult to interpret since DIDS does not inhibit OCT but membrane anion channels, which should not mediate the uptake of the cationic dye PoPo3. It might be that the negatively charged DIDS binds PoPo3 directly, thereby inhibiting binding to DNA and the increase in fluorescence. PoPo3 might be suitable as a surrogate marker for the uptake of chemical compounds/drugs, which is enhanced by CBD. Since RASF produce high amounts of IL-6, IL-8, and MMP-3^60^, we also investigated the impact of CBD on production of these mediators. CBD dose-dependently reduced IL-6, IL-8, and MMP-3 with concomitant reduction in cell viability. CsA was able to rescue RASF from cell death and increased Il-6 and IL-8 production confirming that cell death is the influencing factor on cytokine production.

**Fig. 6:**
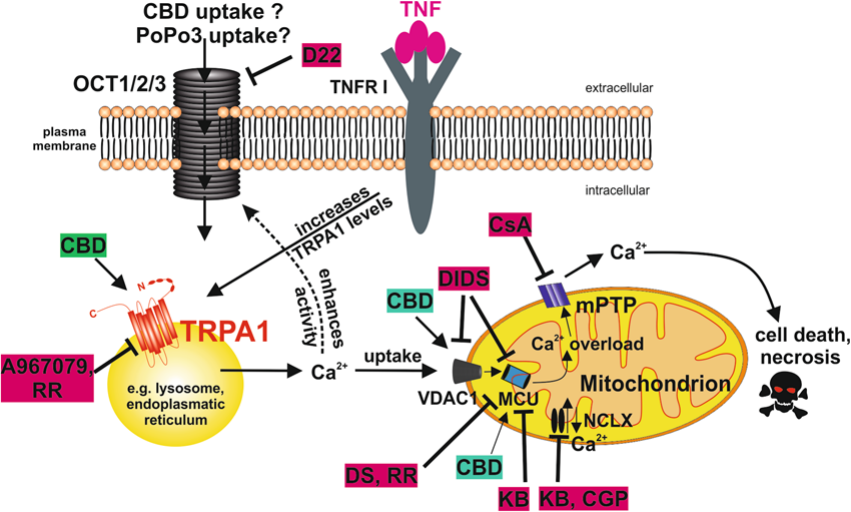
Proposed hypothetical mechanism of CBD-induced cell death. TNF increases TRPA1 protein, which is located in intracellular compartments. CBD activates TRPA1 and Ca^2+^ is released into the cytosol. Elevations in cytosolic Ca^2+^ are reduced through uptake into mitochondria. In addition, increased cytosolic Ca^2+^ might enhance the activity of organic cation transporters (OCT) which might mediate the uptake of the fluorescent dye PoPo3. Additionally, OCT might mediate the uptake of CBD itself. By binding to VDAC1, CBD increases Ca^2+^ flux through the outer mitochondrial membrane. Ca^2+^ is then taken up into the matrix by the mitochondrial Ca^2+^ uniporter (MCU) and, if mitochondria are depolarized, by the Na^+^/Ca^2+^ exchanger (NCLX), which operates in reverse mode under these conditions. Ca^2+^ overload occurs, the mitochondrial permeability transition pore (mPTP) assembles and cell death occurs.

From our data, we propose a mechanism of how CBD influences RASF function and induces cell death under pro-inflammatory conditions (Fig. 6). TNF sensitizes RASF to the action of CBD by up-regulating TRPA1^22^. CBD increases intracellular calcium by activating TRPA1 but it also binds several mitochondrial targets like VDAC1, MCU, and NCLX, which on their part influence cytosolic calcium. Eventually, mitochondrial calcium overload occurs and mPTP is assembled leading to cell death.

CBD has been used in animal model of RA, demonstrating anti-inflammatory and analgesic effects but the mechanism of action has not been identified^17,19^. Here, we demonstrate that CBD reduces cell viability preferentially in TNF-activated RASF via TRPA1 and mitochondrial targets. CBD might decrease chronic inflammation since RA is characterized by a hypoxic environment in the joint with concomitant mitochondrial dysfunction^61^. In this setting, immune cells and RASF might be specifically vulnerable to a “second hit” induced by CBD, leading to deletion of pro-inflammatory immune cells and fibroblasts thereby resolving inflammation. In addition, CBD might also synergize with anti-rheumatic drugs like methotrexate or JAK inhibitors, since it has been reported in tumor cell lines that CBD works in synergy with e.g. the chemotherapeutic drug doxorubicin^62^. Furthermore, CBD also targets TRPV2, which not only increases the uptake of cytotoxic chemotherapeutic agents but also reduces RASF invasion and matrix metalloproteinase production^47,63^. In conclusion, CBD might be beneficial as an adjuvant treatment in rheumatoid arthritis that might support the action of currently used disease-modifying anti-rheumatic drugs.

## Materials and methods

### Biochemicals

#### Patients

In total, 40 patients with long-standing RA fulfilling the American College of Rheumatology revised criteria for RA (24) were included in this study. The RA group comprised of 32 females and 8 males with a mean age of 67.8 years ±10.5 years and 66.9 years ±8.2 years, respectively. C-reactive protein was 47.9 mg/dl ± 186.3 mg/dL for females and 28.7 mg/dL ± 43.2 mg/dL for males and rheumatoid factor was 184.4 iU/mL ± 280.4 iU/mL for females and 31.8 iU/mL ± 37.6 iU/mL for males. IN all, 11 out of 40 patients received glucocorticoids, 7 out of 40 methotrexate, 3 out of 40 biologicals, and 1 out of 40 a JAK inhibitor. All patients underwent elective knee joint replacement surgery, and they were informed about the purpose of the study and gave written consent. The study was approved by the Ethics Committees of the University of Düsseldorf (approval number 2018-87-KFogU) and Regensburg (approval number 15-1 01-021). We confirm that all experiments were performed in accordance with relevant guidelines and regulations (Table 1).

**Table 1:** Biochemicals used in this study.

	order #	vendor	solvent	[c] used in experiments	Reference
DIDS	4523	Tocris	DMSO	50 µM	^32,64^
Cannabidiol	1570	Tocris	DMSO	0.5 µM, 1 µM, 5 µM, 10 µm, 20 µM	^7,8,23^
CGP37157	1114	Tocris	DMSO	1 µM, 10 µM	^30,65^
KB-R7943	1244	Tocris	DMSO	2.5 µM, 25 µM	^55,56^
A967079	4716	Tocris	DMSO	10 µM	^22,66,67^
Ruthenium red	1439	Tocris	H2O	10 µM	^68,69^
Cyclosporin A	30024	Sigma	DMSO	10 µM	^9^
Capsazepine	464	Tocris	DMSO	10 µM	^29,70^
GPN	14634	Cayman	DMSO	250 µM	^29,70,71^
Thapsigargin	1138/1	Tocris	DMSO	10 µM	^72^
Decynium-22	4722	Tocris	DMSO	10 µM	^39,40^

The concentrations used in this study are based on values found in the literature (reference).

#### Synovial fibroblast and tissue preparation

Samples from RA synovial tissue were isolated and prepared as described previously^22^ (for details see also Supplementary methods).

#### Proliferation of RASF

Proliferation was assessed by the cell titer blue viability assay (Promega, Madison, USA, # G8080) according to manufacturer’s instructions.

#### Intracellular calcium and PoPo3 uptake

In black 96-well plates, RASF were incubated with 4 µM of calcium dye Cal-520 (ab171868, abcam, Cambridge, UK) in Hanks buffered salt solution (1 mM Ca^2+^; HBSS, sigma, # 55037 C) or PBS (no Ca^2+^) with 0.02% Pluoronic F127 (Thermo fisher scientific, Waltham, USA, # P6866) for 60 min at 37 °C followed by 30 min at room temperature. After washing, HBSS or PBS containing 1 µM PoPo3 iodide (Thermo fisher scientific, # P3584) and respective antagonists/ligands/inhibitors were added for 30 min at room temperature. After that, CBD was added and the intracellular Ca^2+^ concentration as well as PoPo3 uptake were evaluated with a TECAN multimode reader over 90 min.

#### Flow cytometry

RASF were trypsinized, washed and fixed for 20 min with 3.7% formaldehyde (F8775, Sigma Aldrich). Cells were permeabilized with 0.1% Triton X-100 (X100, Sigma) in PBS for 10 min. Then, 0.2 µg/50 µl primary antibody (Proteintech, 19124-1-AP) was added for 2 h. The secondary antibody (Abcam, goat anti-rabbit IgG H&L (Alexa Fluor® 488), ab150077) was incubated for 1 h. Cells were analyzed using a MACS Quant 9 analyzer (Miltenyi Biotec, Bergisch Gladbach, Germany).

#### RealTime-Glo cell viability assay

Cell viability was assessed according to manufacturer’s instructions (Promega, # G9711).

#### Statistical analysis

Statistical analysis was performed with SPSS 25 (IBM, Armonk, USA). The statistic tests used are given in the figure legends. Normal distribution was determined using the Shapiro–Wilk test, equal variance was determined by Levene’s test. In the case of equal variance, the Bonferroni post-hoc test was used, otherwise the Dunnet’s post-hoc test was employed. When data are presented as box plots, the boxes represent the 25th to 75th percentiles, the lines within the boxes represent the median, and the lines outside the boxes represent the 10th and 90th percentiles. When data are presented as line plots, the line represents the mean. When data are presented as bar charts, the top of the bar represents the mean and error bars depict the standard error of the mean (sem). The level of significance was *p* < 0.05.

## Supplementary information

supplementary results and methods

supplementary figure 1

supplementary figure 2

supplementary figure 3

## Data Availability

The datasets used and/or analysed during the current study are available from the corresponding author on reasonable request.
