# Supplementary material for: Cannabidiol (CBD): a killer for inflammatory rheumatoid arthritis synovial fibroblasts
Source: Cell Death Dis. 2020 Sep 1;11(8):714. doi: 10.1038/s41419-020-02892-1 (PMC7463000; doi:10.1038/s41419-020-02892-1)
Supplement: Supplementary file 1 — supplementary results and methods [file 41419_2020_2892_MOESM1_ESM.docx]

**Suppl. Results:**

We employed KB-R7943 (KB, 2.5µM and 25µM) and CGP37157 (1µM and 10µM), which are ligands for NCLX and DSDS16570511, which ligates the MCU (suppl. Fig. 2). While CGP37157 blocks transport of calcium out of the mitochondrial matrix, KB-R7943 is a reverse mode inhibitor that inhibits calcium transport into the matrix. However, the effects of the latter are concentration-and cell type specific and in some cases KB acts as a forward mode inhibitor as well ^37^. Furthermore it has been discovered that KB also blocks the MCU, which is the major protein facilitating calcium entry into the mitochondrial matrix ^38^
KB-R7943 increased basal calcium levels in unstimulated RASF (suppl. Fig.2E, I). TNF pre-incubation did only slightly modulate the calcium response to 10µM CBD at 25µM but not at 2.5µM KB (suppl. Fig.2J). Without TNF pre-stimulation, PoPo3 uptake was enhanced by KB (2.5µM) when CBD was added at 10µM and 20µM (suppl. Fig. 2G), while it reduced PoPo3 uptake under TNF pre-stimulated conditions (suppl. Fig. 2H). Interestingly, at 25µM KB not only enhanced basal uptake of the dye (suppl. Fig. 2K, blue line), but also increased the CBD-induced uptake at 5µM and 20µM CBD in TNF pre-treated RASF (suppl. Fig. 2L). Next, we investigated the effects of CGP37157 (CGP) a forward mode inhibitor of the NCLX, which decreases the export of calcium from the mitochondrial matrix ^39^. Intracellular calcium levels were unaltered by CGP in unstimulated RASF after CBD challenge. TNF pre-incubation revealed an inhibitory effect of CGP on CBD-induced calcium elevations, which was most pronounced at 10µM CGP (suppl. Fig. 2R). PoPo3 uptake was slightly enhanced under unstimulated conditions by 1µM CGP and 0µM or 20µM CBD (suppl. Fig. 2O). At 10µM, the effects were increased and CGP decreased PoPo3 uptake induced by 10µM and 20µM CBD (suppl. Fig. 2S). TNF pre-stimulation changed the effects of CGP on PoPo3 uptake from slightly inhibitory to stimulatory. At 1µM, CGP enhanced PoPo3 uptake in TNF pre-stimulated RASF at 5µM, 10µM and 20µM CBD (suppl. Fig. 2P), while 10µM CGP only enhanced the response to 20µM CBD (suppl. Fig. 2T). Of note, without the addition of CBD, KB (2.5µM and 25µM) decreased basal calcium levels and PoPo3 uptake regardless of TNF pre-stimulation. In contrast, CGP increased intracellular calcium (CGP: 10µM) and PoPo3 uptake (CGP: 1µM and 10µM) in TNF pre-treated RASF (suppl. Fig. 3A-D).
The cytotoxic effects of CBD have already been inhibited by blocking the mitochondrial calcium uniporter (MCU) ^25^ and, therefore, we also investigated the contribution of this transporter. Calcium levels and PoPo3 uptake were reduced by the MCU inhibitor DS16570511 (DS) ^44^ in unstimulated RASF at 10µM and 20µM CBD (suppl. Fig. 2U, W). When RASF were pre-treated with TNF, intracellular calcium (suppl. Fig. 2V) and PoPo3 uptake (suppl. Fig. 2X) were enhanced. In addition, basal calcium levels and PoPo3 uptake were increased by DS (suppl. Fig. 3A-D).

**Suppl. methods:**

**XCELLigence assay**

The XCELLigence system (ACEA biosciences, San Diego, USA) analyzes cellular impedance on gold electrode covered 96 well plates. Changes in resistance/impedance occur by cell morphologic changes and are correlated with cell viability. 10.000 RASF/well were seeded and stimulated with CBD on the next day. Impedance changes were monitored over 1h and were quantified using the cell index.

**Data representation in calcium assays**

Data were normalized twice: 1. Normalization within one sample. The first four readings were recorded before addition of CBD and the mean served as a baseline and was set to 100%. 2. Normalization to control. Data shown in figures 2-5 are depicted as % of the unstimulated (vehicle) control. For this, all readings within the control were set to 100% at each time point and all results presented here are shown as % of control.

**Stimulation of RA synovial fibroblasts**

6000 cells were seeded onto 96 well microtiter plates, grown until 80-90% confluency was reached and then stimulated with either TNF (10ng/mL, peprotech, #300-01A) or left untreated for 72h. After that, cells were used for the determination of intracellular calciumlevels, PoPo3 uptake, Real-Time Glo or proliferation assays.

**Synovial fibroblast and tissue preparation**

Samples from RA synovial tissue were collected and transferred in serum-free RPMI 1640 medium immediately after opening the knee joint capsule. On the same day, tissue was prepared for cell isolation (25). Synovial tissue was cut into small fragments and treated with liberase (50µg/mL) (Roche Diagnostics, Mannheim, Germany) at 37°C overnight. The cell suspension was filtered (70 µm) and centrifuged at 300 g for 10 min. Pelleted cells were treated with erythrocyte lysis buffer (20.7 g NH4Cl, 1.97 g NH4HCO3, 0.09 g EDTA ad 1 l H2O) for 5 min. After addition of an equal volume of RPMI 1640 medium with 10% FCS, cells were recentrifuged for 10 min and resuspended in RPMI-1640 (Sigma Aldrich, St. Louis, USA) with 10% FCS afterwards. After overnight incubation, RPMI medium was replaced with fresh medium to wash off dead cells and debris.

**Determination of IL-6, IL-8 and MMP-3**

Cytokines in RASF supernatants were quantified using commercial ELISAs for IL-6, IL-8 (OptEIA, New Jersey, USA) and total MMP-3 (R&D systems, Minneapolis, USA) after the suppliers instructions.

**RealTime-Glo data presentation**

In experiments shown in Fig. 1D and 1E, CBD was added together with RealTime-Glo (continuous-read method with reagent addition at test compound dosing). In subsequent experiments (Fig. 1G-K) we established a baseline luminescence. For this purpose, we incubated RASF with RealTime-Glo reagent for 2h. Then, respective antagonists (A967079, Ruthenium Red, Cyclosporin A, DIDS) were added for 30min. After that, CBD was added. Luminescence was recorded after the antagonists were added. Due to the differential addition of compounds, curves in 1D, E show an increase at the beginning, whereas in 1G-K, data acquisition started right an already elevated luminescence level. All results were normalized to the control, which was set to 100% at time point 2h.

Suppl. Fig. 1:

**Cell morphologic changes induced by CBD detected by the XCelligence system**. Unstimulated RASF were treated with CBD over 1h. The decline in cell index (CI) corresponds with cell morphologic changes. The greater the coverage of cells within a microwell, the higher the CI. Decreases in CI are associated with rounding and detachment of cells as it occurs during necrosis or apoptosis. ANOVA with Bonferroni post-hoc test was used for comparisons. ***p<0.001

Suppl. Fig. 2:

**Modulation of intracellular calcium levels and PoP3 uptake of RASF after CBD challenge with concomitant inhibition of mitochondrial targets**. Intracellular Ca^2+^ levels (A, B, E, F, I, J, M, N, Q, R, U, V) and PoPo3 uptake (C, D, G, H, K, L, O, P, S, T, W, X) of RASF after CBD challenge and concomitant inhibition with KB-R7943 mesylate (KB) (E-L), CGP37157 (CGP) (M-T) or DS16570511 (U-X). n is the number of experiment replicates from 24 patient samples (A, C), 33 patient samples (B, D) and 8 patient samples (N, P, R, T). E-X (except N, P, R, T) n number equals number of replicates and different patient samples. ANOVA with Dunnett’s post-hoc test was used for all comparisons versus A, B, C or D. *p<0.05, **p<0.01, ***p<0.001

Suppl. Fig. 3:

**Influence of antagonists used in this study on basal Ca^2+^ levels and PoPo3 uptake**. A, C) Intracellular Ca^2+^ levels and B, D) PoPo3 levels after 30min incubation with given inhibitors before the addition of CBD. n is the number of data points included in the analysis. Before CBD addition, 4 baseline readings were conducted in each microplate well and all baseline readings per condition were put together and are depicted as box plots. ANOVA with Bonferroni correction was used for all comparisons versus the respective controls (with or without TNF pre-stimulation, no inhibitor added).*p<0.05, **p<0.01, ***p<0.001. ### indicates the difference between TNF pre-stimulated samples and unstimulated RASF.
