# Supplementary figures and images for: Cannabidiol (CBD): a killer for inflammatory rheumatoid arthritis synovial fibroblasts
Source: Cell Death Dis. 2020 Sep 1;11(8):714. doi: 10.1038/s41419-020-02892-1 (PMC7463000; doi:10.1038/s41419-020-02892-1)

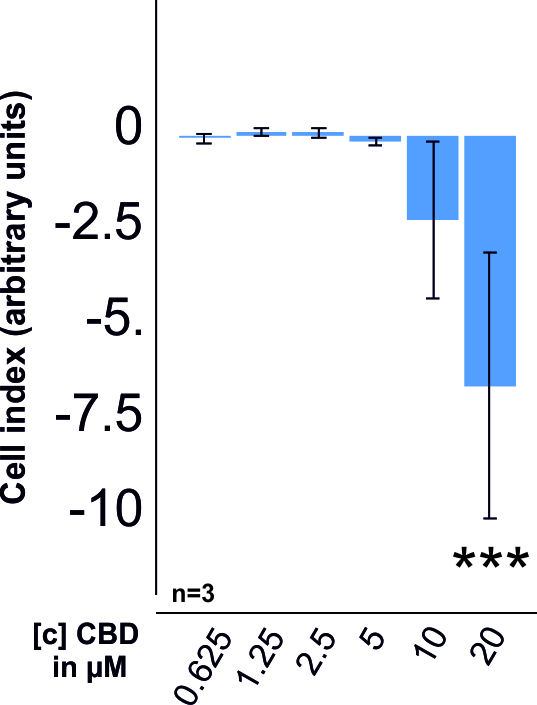

Supplement: Supplementary file 2 — supplementary figure 1 [file 41419_2020_2892_MOESM2_ESM.tif]

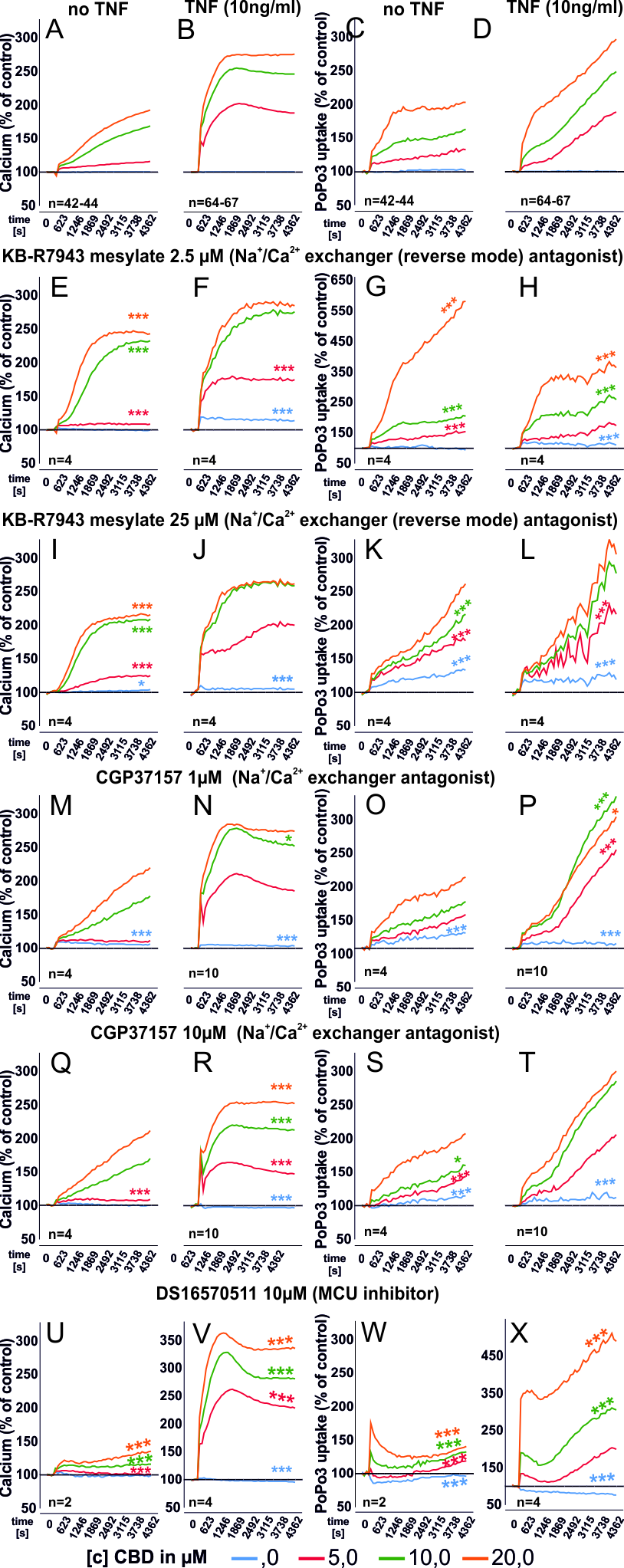

Supplement: Supplementary file 3 — supplementary figure 2 [file 41419_2020_2892_MOESM3_ESM.tif]

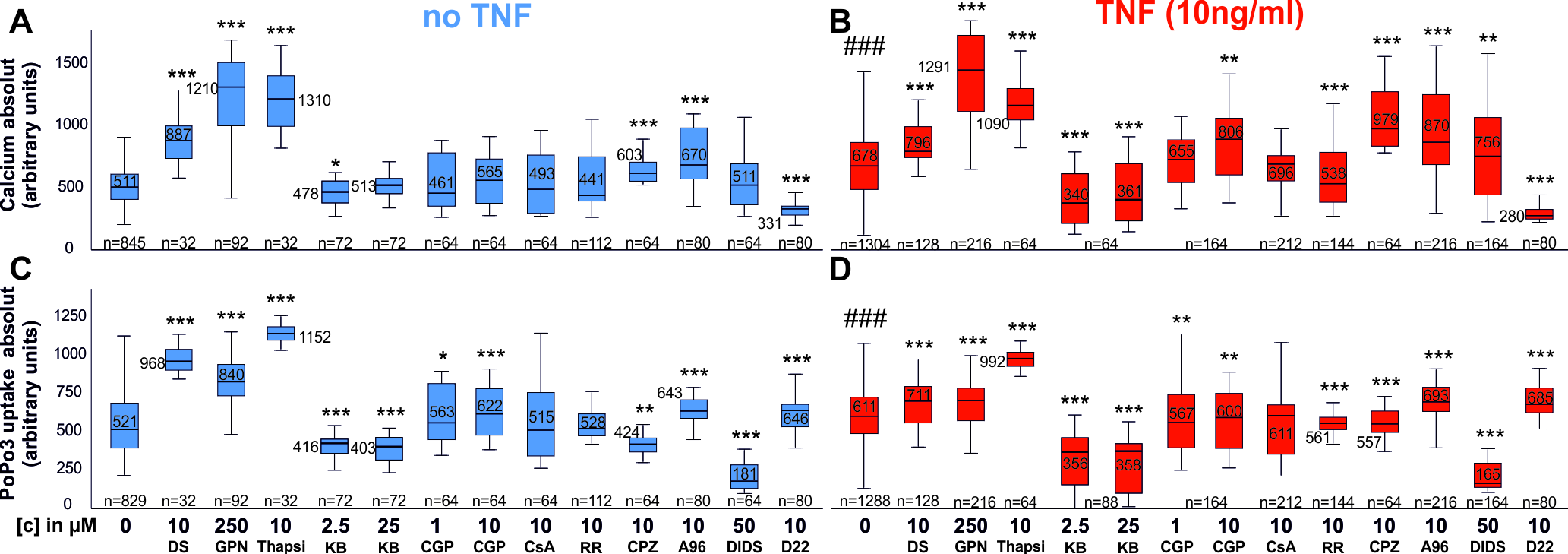

Supplement: Supplementary file 4 — supplementary figure 3 [file 41419_2020_2892_MOESM4_ESM.tif]
